# Cadmium Exposure and Risk of Any Fracture: A PRISMA-Compliant Systematic Review and Meta-Analysis

**DOI:** 10.1097/MD.0000000000002932

**Published:** 2016-03-11

**Authors:** Xianlin Cheng, Yuming Niu, Qingyang Ding, Xinhai Yin, Guanglei Huang, Juxiang Peng, Jukun Song

**Affiliations:** From the Department of Oral and Maxillary Surgery (XC, XY, GH, JS), Gui Zhou Provincial People's Hospital, Guiyang; Department of Stomatology and Center for Evidence-Based Medicine and Clinical Research (YN), Taihe Hospital, Hubei University of Medicine, Shiyan; Department of Oral Medicine (QD), Chengdu Yafei Dental Dashijie Clinic, Chengdu; and Department of Orthodontics (JP), Stomatology Hospital of Guiyang, Guiyang, China.

## Abstract

Several observational studies have investigated the relation between cadmium exposure and risk of any fracture. However, the results from epidemiological studies for the association are inconsistent.

We conducted a meta-analysis to evaluate the relationship between cadmium exposure and risk of any fracture. The pertinent studies were identified by a search of PubMed and Embase databases from 1966 to June 2015.

Seven articles involving 21,941 fracture cases and 504,346 participants were included. The meta-analysis showed that the pooled relative risk of any fracture for the highest versus lowest category of cadmium concentration was 1.30 (95% confidence interval = 1.13–1.49). In subgroup analyses, the significant association remained consistent when stratified by study type, geographical region, method of cadmium exposure assessment, and gender.

Our meta-analysis showed that a high cadmium exposure may be a risk factor for any fracture. However, this result should be interpreted cautiously because of the heterogeneity among studies and existence of publication bias. Additional large, high-quality prospective studies are needed to evaluate the association between cadmium exposure and the risk of development of fracture.

## INTRODUCTION

Cadmium (Cd) is a persistent heavy metal with high toxicity and an elimination half-life of 10 to 30 years. Cd may have a wide range of negative effects on human health.^1^ In general nonsmoking population, major sources of Cd exposure are smoking and diet because tobacco, grains, potatoes, and vegetables take up Cd from soil.^2^ In a public health context, the negative effect of Cd on bone in the general population is of great concern following the outbreak of Itai-itai disease in Japan more than 50 years ago.^3^ Recently, numerous observational studies were conducted to evaluate the effects of Cd exposure on any fracture risk. However, the results of those studies are inconsistent, with majority of the studies reporting positive effects,^4–8^ whereas other studies found mixed results.^9–11^ In addition, these studies have a modest sample size, and the magnitude of the association is variable among these studies, with relative risk (RR) varying from 0.99 (95% confidence interval [CI]: 0.93–1.05) to 4.10 (95% CI = 1.55–6.61), and thus the magnitude is limited by the low precision in risk estimates. These epidemiological studies lack comprehensive assessment of Cd exposure. Given the popularity of Cd exposure and poor prognosis of fracture, any risk factors for the development of fracture would have a substantial impact on public health. Therefore, we systematically performed a meta-analysis by combining all available data of observational studies to evaluate the association between Cd exposure and risk of fracture.

## MATERIALS AND METHODS

The study was reported following the Preferred Reporting Items for Systematic Reviews.^12^ No ethical issues were involved in our study given that our data were based on published studies.

### Literature Search

We identified studies examining the relation between Cd exposure and any fracture risk by systematically searching the database of PubMed and Embase for papers published from 1966 to June 20, 2015. The predefined keywords were used without any limitation: “fracture(s)” combined with “cadmium.” Furthermore, we reviewed the reference lists from retrieved articles for additional relevant studies.

### Eligibility Criteria and Study Selection

Studies were considered acceptable for inclusion in the meta-analysis if they met the following criteria: study design was either cohort, case–control, or cross-sectional; the exposure was Cd exposure; the outcome was fracture risk; and hazard ratio (HR) or RR and odds ratio (OR), with corresponding 95% CI (or data to calculate these) were reported. Studies were excluded if they met the following criteria: they are editorial letters, historical reviews, and descriptive studies, such as case reports and case series, or laboratory studies; they did not contain enough data for calculating RR; and when multiple publications covered the same study population, only the study with the larger sample was included. Two authors (JKS and YXH) independently evaluated the eligibility of all retrieved studies and disagreements were resolved through discussion or consultation with a third author (HGL).

### Data Extraction

Two authors (JKS and YXH) independently extracted data from the selected studies. The following data were extracted: first author, publication year, study design, country, sex, total number of cases and subjects, assessment methods for Cd exposure, and adjusted variables. The adjusted RR was extracted in preference to the nonadjusted RR; however, the unadjusted RR and CI were calculated when the RR was not provided. When more than 1 adjusted RR was reported, the ratio with the most number of adjusted variables was selected. Any disagreements were resolved through discussion and consensus.

### Quality Evaluation

We evaluated the methodological quality of included studies using the Newcastle–Ottawa scale (NOS).^13^ The check list contains 9 items for case–control studies and cohort studies with every item accounts for 1 point. We allocated high-quality studies with a score >5.

### Statistical Analysis

We used the RR with 95% CI as a common measure across all eligible studies. The differences among risk estimates (HR, RR, and OR) were ignored and the OR and HR were directly converted to RR because fracture is a relatively rare event. A random-effects model of the DerSimonian and Laird method, which is appropriate when the heterogeneity cannot readily be explained, was used to calculate summary RR comparing the highest versus lowest level of Cd across all included studies regardless of heterogeneity.^14^ If sex-specific estimates were available, then they were also regarded as 2 different studies.^7–9^

Given that patient characteristics, study design, and other confounding factors were inconsistent among studies, sensitivity analysis was performed to evaluate robustness and stability by sequentially omitting 1 study on each turn. Moreover, subgroup analyses were subsequently carried out by study type, geographical region, method of Cd exposure assessment, gender, and fracture site. We evaluated the potential publication bias using a funnel plot and Egger tests, with *P* < 0.1 indicating significant publication.^15^ All statistical analyses were carried out using Stata version 13.1 (Stata Corp., College Station, TX).

## RESULTS

### Literature Search

**FIGURE 1:**
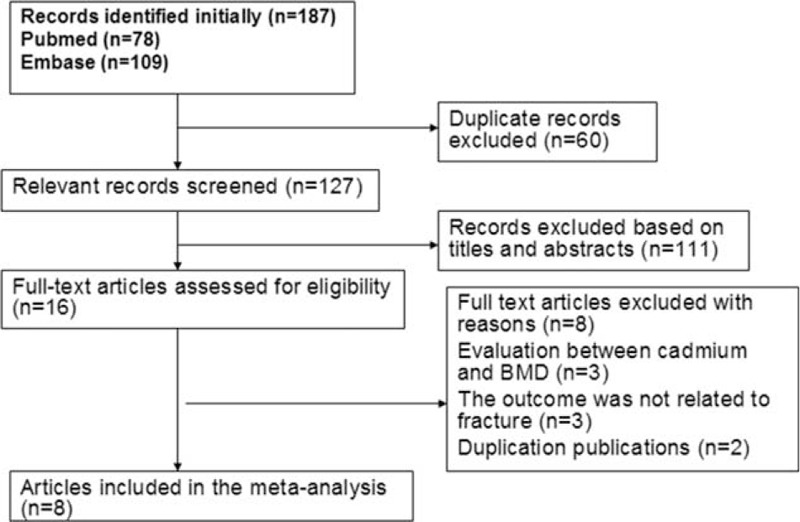
Flow diagram of systematic literature search.

A diagram showing the details of study inclusion is shown in Figure 1. The search strategy yielded 187 citations, of which 60 studies were excluded because they were duplicate publications; 127 studies were initially screened and 111 studies were excluded based on their titles and abstracts. We reviewed the 16 possible relevant articles in full-text. Three articles reporting relation between Cd exposure and bone mineral density (BMD) were excluded,^16–18^ 3 articles were also excluded because the outcome was unrelated to fracture,^19–21^ and 2 articles were excluded because they covered the same population.^22,23^ Two articles covered the same population, but the articles employed different biomarkers to evaluate the level of Cd exposure.^4,5^ Finally, 8 articles^4–11^ were considered eligible in the meta-analysis.

### Study Characteristics and Quality Assessment

Individual characteristics of the included 8 articles (6 cohort studies and 2 case–control articles) were summarized in Table 1 . They were published from 1999 to 2014. Of the 8 articles, 7 studies were from the Europe,^4–6,8–11^ and 1 study was from China.^7^ Three articles were designed to evaluate the association between Cd exposure and hip fracture risk,^6,8,11^ and 4 articles evaluated the relation between Cd exposure and any fracture risk,^5–7,9^ 2 articles evaluated the association between Cd exposure and forearm fracture.^4,10^ Four articles used urinary Cd (U-Cd) as biomarkers for long-term exposures to Cd,^4,7,9,10^ whereas 2 articles evaluated the Cd exposure levels by estimating the dietary Cd (D-Cd) using food frequency questionnaire^5,6^; 1 article examined the Cd exposure levels in drinking water,^8^ and 1 article investigated the Cd exposure levels in the erythrocytes (Ery-Cd).^11^ One article combined the assessment of dietary and U-Cd in relation to any fracture.^5^ Two studies were designed to evaluate the relationship between D-Cd exposure and fracture risk among nonsmoker populations.^4,6^ Six articles adjusted for a group of conventional risk factors for fracture,^4–6,8–11^ whereas 1 article did not control for other confounding factors,^7^ only 3 articles adjusted for smoking status.^5,9,10^ As shown in the Table 2, the quality scores ranged from 4 to 6, with mean of 5.

**TABLE 1:**
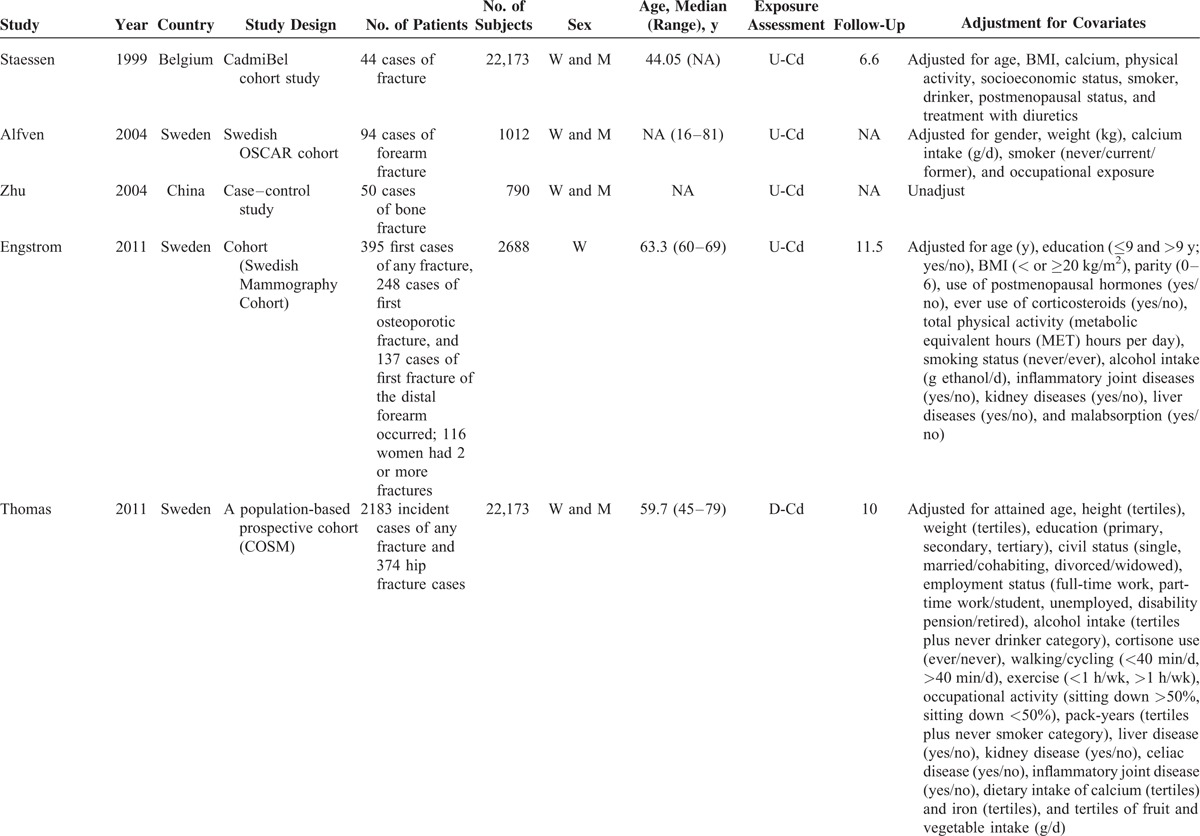
Characteristics of Studies Included in the Meta-Analysis

**TABLE 1 (Continued):**
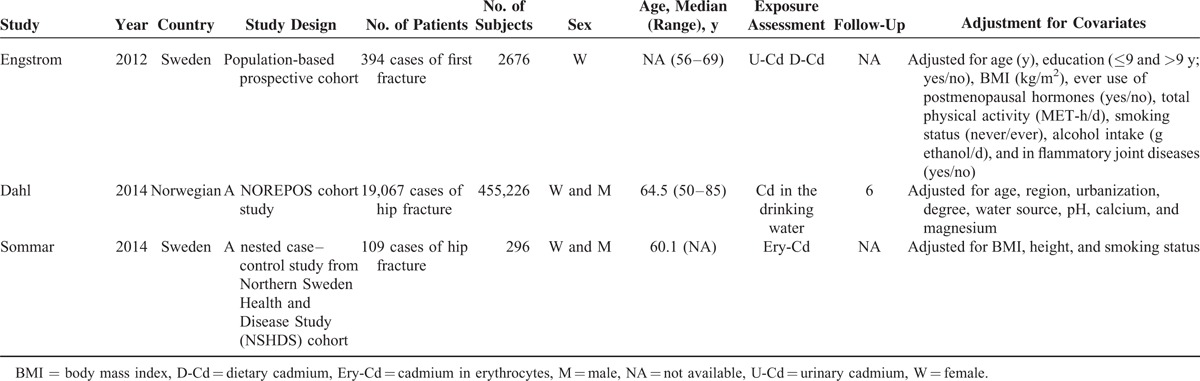
Characteristics of Studies Included in the Meta-Analysis

**TABLE 2:**
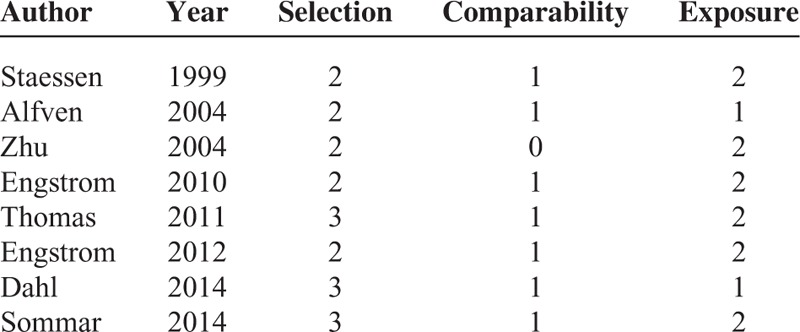
Quality Assessment of Eligible Studies Based on Newcastle–Ottawa Scale

### Quantitative Synthesis

**FIGURE 2:**
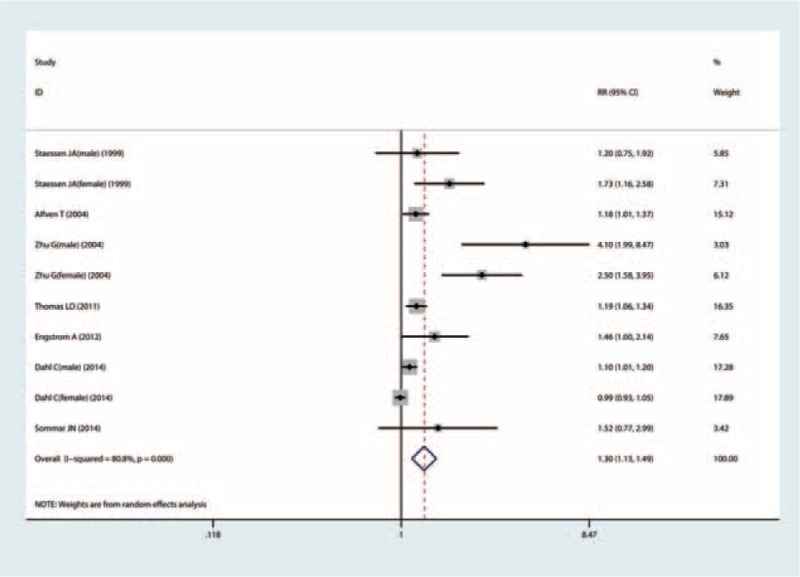
Forest plot for the association between cadmium exposure and any fracture risk.

A total of 10 studies from 7 articles^5–11^ with 22,336 cases and 507,034 participants were included, because 3 results (for male and female) were reported in 3 publications.^7–9^ The overall summary RR for fracture was 1.30 times (RR = 1.30; 95% CI = 1.13–1.49) for the highest category of Cd exposure compared with the lowest category, with significant heterogeneity (*P* of heterogeneity = 0.000, *I*^2^ = 80.8%; Figure 2; Table 3).

**TABLE 3:**
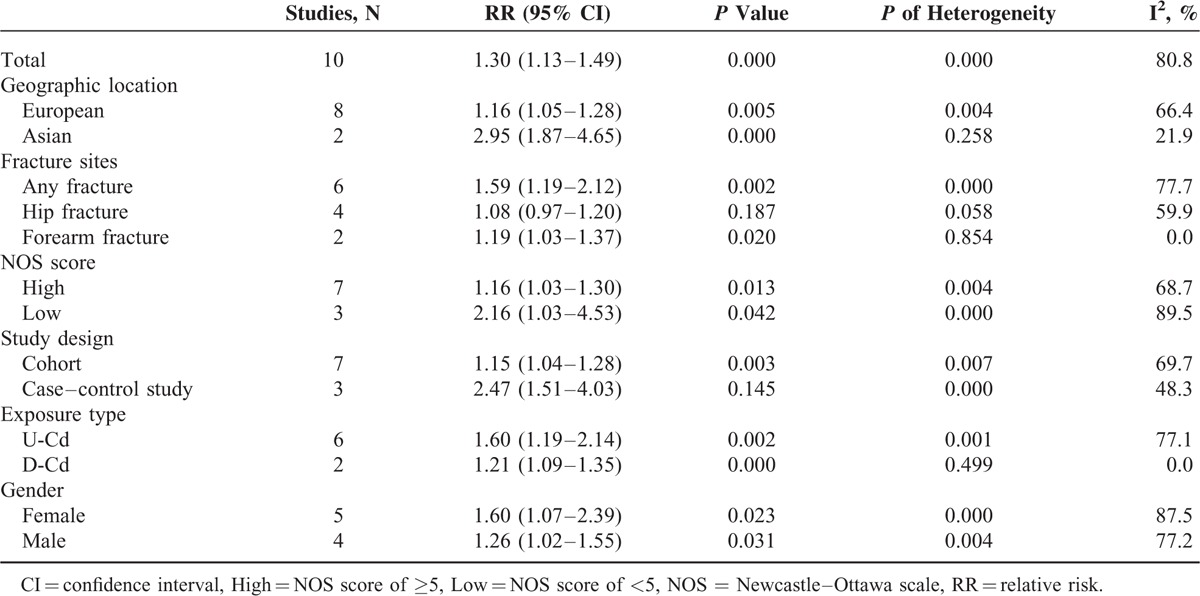
Results of Overall Subgroup Analysis

### Sensitivity Analysis and Subgroup Analyses

**FIGURE 3:**
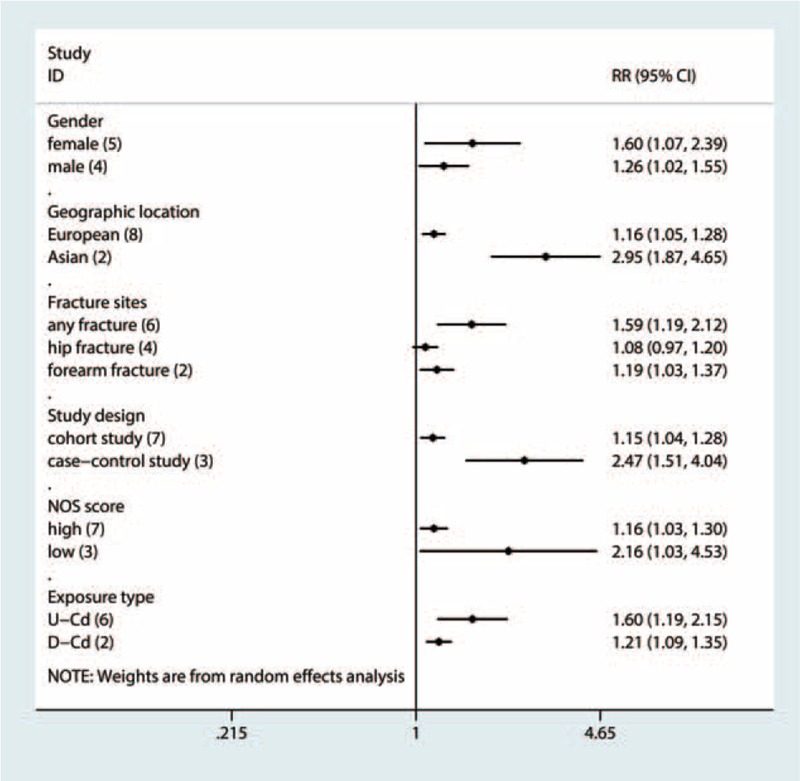
Subgroup analyses for the association between cadmium exposure and any fracture risk.

To test the robustness and stability of the relation, we performed sensitivity analyses to omit 1 study at a time and to compute the combined RR for the remaining studies. The combined RR for fracture ranged from 1.22 (95% CI = 1.08–1.38) to 1.40 (95% CI = 1.16–1.68) in the sensitivity analyses after excluding 1 study at a time. In the sensitivity analysis, similar results were observed, which ranged from 1.22 (95% CI = 1.08–1.38) with low heterogeneity (*I*^2^ = 76.4%, heterogeneity *P* = 0.000) (excluding the study by Zhu et al^7^) to 1.40 (95% CI = 1.16–1.68) with significant heterogeneity (*I*^2^ = 82.9%, heterogeneity *P* = 0.000) (excluding the study by Dahl et al^8^). Furthermore, subgroup analysis was also performed (Figure 3; Table 3). In subgroup analyses for study design, the summary RR values of any fracture for the highest category of Cd exposure versus lowest category were 2.47 (95% CI = 1.51–4.03) and 1.15 (95% CI = 1.04–1.28) for the 2 case–control studies and 5 cohort studies, respectively. When stratified by method of Cd assessment, we found an increase of any fracture risk in both U-Cd (RR = 1.60, 95% CI = 1.19–2.14) and D-Cd exposure (RR = 1.21, 95% CI = 1.09–1.35). The combined RR for any fracture was 1.16 (95% CI = 1.05–1.28) for studies conducted in Europe and 2.95 (95% CI = 1.87–4.65) for that in Asia. When stratified by fracture site, Cd exposure significantly increased the risk of forearm fracture (RR = 1.19, 95% CI = 1.03–1.37) and any fracture risk (RR = 1.59, 95% CI = 1.19–2.12), but not the risk of hip fracture (RR = 1.08, 95% CI = 0.97–1.20). Compared with the low NOS score (OR = 2.16, 95% CI = 1.03–4.53), the association remained significant among studies with high NOS score (OR = 1.16, 95% CI = 1.03–1.30). When stratified by gender, we found an increase of any fracture risk in both male (OR = 1.26, 95% CI = 1.02–1.55) and female (OR = 1.60, 95% CI = 1.07–2.39). Only 2 studies evaluated the effect of D-Cd exposure and risk of any fracture among nonsmoker populations; the overall RR was 1.30 (95% CI = 1.05–1.61), with low heterogeneity (*I*^2^ = 17.1%, heterogeneity *P* = 0.272). For the 4 studies adjusted for smoking status, the pooled RR was 1.30 (95% CI = 1.10–1.54), with low heterogeneity (*I*^2^ = 20.3%, heterogeneity *P* = 0.288).

### Publication Bias

**FIGURE 4:**
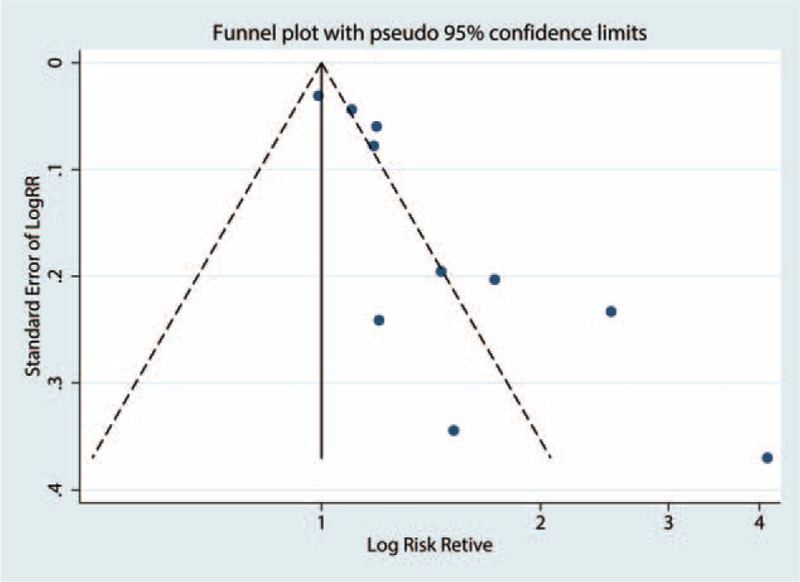
Funnel plot of Cd exposure and any fracture risk.

Some asymmetry was observed in the funnel plot (Figure 4), with *P* values of 0.074 for Begg test and 0.001 for Egger test, suggesting the existence of publication bias.

## DISCUSSION

To the best of our knowledge, this article presents the first meta-analysis to explore the role of exposure to Cd in patients with any form of fracture. The overall results of the present meta-analysis using a random-effects model provide evidence that a high Cd exposure may be a risk factor for increased risk of any fracture. The pooled estimates were robust according to sensitivity and subgroup analyses.

Cadmium is widely distributed in the environment through industrial and agricultural activities. A prospective cohort of CadmiBel study in Belgium found a higher risk of any fractures in women (RR = 1.73, 95% CI = 1.16–2.57) and a nonsignificant increase in risk of any fractures in men (RR = 1.20, 95% CI = 0.75–1.93) with a doubling of U-Cd.^9^ Similarly, a cohort study conducted in Sweden found an 18% (95% CI = 1.01–1.37) increase in risk of forearm fracture per unit increment in U-Cd (nmol Cd/mmol creatinine) in those over 50 years of age, but nonsignificant in those below 50 years old.^10^ A significant association between U-Cd exposure and any fracture risk was found among women and men in a case–control study conducted in China^7^ and in a population-based prospective cohort.^6^ In a same cohort in Sweden, the combination of the 2 biomarkers, U-Cd and D-Cd, showed Cd exposure increased the risk of any fracture in the general population.^4,5^ In a Norwegian cohort study, a considerably low level of Cd in drinking water was positively associated with increased risk of any fracture.^8^ In another cohort study in Sweden, Sommar et al^11^ examined the effect of Cd in erythrocytes on risk of any fracture and found positive association among women, but not among men. In the present meta-analysis, we found a positive association between Cd exposure and risk of any fracture.

Results from subgroup analyses indicated that geographic region, study design, NOS scale, gender, fracture type, and method of Cd assessment are potential sources of heterogeneity. Despite intrinsic limitations of observational study, some results from subgroup analyses remain notable. When stratified by gender, the association remained significant for both male and female. Given that Cd concentrations in urine and whole blood are the most common biomarkers for Cd exposure, U-Cd mainly reflects Cd accumulation in the kidney, which is determined by lifelong exposure, whereas D-Cd and whole-blood Cd demonstrate a combination of both current and historical exposure. Results from subgroup analyses stratified by assessment of Cd exposure showed that both U-Cd and D-Cd were associated with increased risk of any fracture. Smoking is a primary source of exposure in the general population. In addition, smoking is known to cause reduced BMD and may act on bone both directly and indirectly. Thus, we also performed subgroup analyses among nonsmokers to minimize any possible non-Cd-mediated negative effects of tobacco smoking on bone. Cadmium exposure was related to increased risk of any fracture among nonsmoker population (RR = 1.30, 95% CI = 1.05–1.61). Only 3 publications adjusted for smoking status, and the results showed that Cd exposure is associated with increased risk of any fracture (OR = 1.30, 95% CI = 1.10–1.54), with low heterogeneity (*I*^2^ = 20.3%, *P* for heterogeneity = 0.288).

The mechanisms for Cd-induced bone effects remain unclear. Several studies using in vivo animal studies demonstrated that Cd exposure can negatively impact the bone health.^24–27^ The negative effects on bone health are considered to be mediated by indirect renal damage and/or a result of a direct effect on the skeleton.^28^ Experimental data show a direct effect of Cd on bone with decreased bone formation and increased bone resorption at Cd concentrations related to human exposures.^17,28^ Cadmium may interfere with the metabolism of calcium, vitamin D, and collagen, and bone disorders such as osteomalacia or osteoporosis are late manifestations of severe Cd poisoning.^29^ Nevertheless, the associations between Cd exposure and kidney tubular damage and osteoporosis support a kidney-mediated indirect effect.^16,30^

The present meta-analysis has several strengths. First, to the best of our best knowledge, no meta-analysis of the relation between Cd exposure and any risk of fracture has been published. Second, the large number of total cases provided high statistical power for quantitative assessment of the association between Cd exposure and risk of any fracture. Third, the meta-analysis used U-Cd and D-Cd as biomarkers of Cd, which indirectly reflected Cd concentrations; both U-Cd and D-Cd exposures were positively associated with increased risk of any fracture. Fourth, the association of Cd exposure with risk of any fracture remains statistically significant according to sensitivity and subgroup analyses, which indicated that our main findings are robust and Cd exposure may be independent of conventional risk factors of any form of fracture.

Nevertheless, some limitations should be considered in the present meta-analysis. First, observational studies have inherent limitations, such as selective bias and recall or memory bias. Therefore, the findings should be interpreted with caution. Second, the evidence of publication bias was observed; the results could be biased by the publication bias because studies with small sample size and null results may have been rejected for publication. Third, the small number of studies included in the meta-analysis limits the ability to draw a reliable conclusion, especially in the subgroup analyses. Fourth, between-study heterogeneity is common in the meta-analysis, and exploring the potential sources of between-study heterogeneity is essential. Our sensitivity and subgroup analyses indicated the existence of heterogeneity. Fifth, given that the included studies used different methods to assess and categorize Cd exposure, our findings are likely influenced by the misclassification of exposure. Moreover, the potential for misclassification of exposure to Cd may contribute to the heterogeneity among all studies in the summary analysis. Therefore, this result should be considered with caution because of exposure misclassification. In general, the aforementioned limitations may affect our final conclusions.

In summary, the current meta-analysis demonstrates that a high Cd exposure may be a risk factor for any fracture. However, this result should be interpreted cautiously because of the heterogeneity among studies and existence of publication bias. Additional large, high-quality prospective studies are needed to evaluate the association between Cd exposure and the risk of development of fracture.
